# Prescribing for the Eye

**Published:** 1923-06

**Authors:** 


### Prescribing for the Eye.

SIR,—If chemists are not allowed to prescribe medicines, why should opticians be allowed to prescribe glasses? The optician is not a trained specialist, yet a person who feels that his sight needs help has only to walk into an optician's shop to be fitted out with glasses which he is assured will do all that is necessary. But suppose, for instance, that the applicant is suffering from glaucoma. He does not know it—neither does the optician—and it is obvious that in such a case the gravest harm may be done. The optician may be a useful tradesman; he is assuredly not an oculist. The best opticians refuse to act except upon a medical prescription, and it is strange indeed that, while we safeguard teeth, we leave eyes, which are infinitely more important, to the mercies of anybody who likes to call himself an optician.

A LAYMAN.

